# Impact of a complex gender-transformative intervention on maternal and child health outcomes in the eastern Democratic Republic of Congo: protocol of a longitudinal parallel mixed-methods study

**DOI:** 10.1186/s12889-019-8084-3

**Published:** 2020-01-14

**Authors:** Wyvine Ansima Bapolisi, Giovanfrancesco Ferrari, Clara Blampain, Jean Makelele, Lenneke Kono-Tange, Ghislain Bisimwa, Sonja Merten

**Affiliations:** 1 Swiss Tropical and Public Health Institute, Basel, Switzerland; 2 University of Basel, Basel, Switzerland; 3 Université Catholique de Bukavu, Bukavu, Democratic Republic of the Congo; 4 CARE International DRC, Kinshasa, Democratic Republic of the Congo; 5 CARE Nederland, The Hague, Netherlands

**Keywords:** Women’s empowerment, Decision-making, Gender equality, Male involvement, SGBV, Child nutritional status, Reproductive health, Family planning

## Abstract

**Background:**

In the eastern part of the Democratic Republic of Congo (DRC) Village Savings and Loan Associations (VSLAs) programs targeting women are implemented. In the context of the ‘Mawe Tatu’ program more equitable intra-household decision-making is stipulated by accompanying women’s participation in VSLAs with efforts to engage men for more gender equality, expecting a positive effect of this combined intervention on the household economy, on child nutritional status, on the use of reproductive health services including family planning, and on reducing sexual and gender-based violence (SGBV).

**Methods:**

A longitudinal parallel mixed method study is conducted among women participating in VSLAs in randomly selected project areas and among a control group matched for socioeconomic characteristics. Descriptive statistics will be calculated and differences between intervention and control groups will be assessed by Chi2 tests for different degrees of freedom for categorical data or by t-tests for continuous data. Structural equation modelling (SEM) will be conducted to investigate the complex and multidimensional pathways that will affect household economic status, child nutritional status and use of reproductive health services. Analysis will be conducted with STATA V.15.

Concomitantly, qualitative data collection will shed light on the intra-household processes related to gender power-relations that may be linked to women’s participation in economic activities and may lead to improvements of maternal and child health. Focus group discussions and in-depth interviews will be conducted. All narrative data will be coded (open coding) with the help of qualitative data analysis software (Atlas TI).

**Discussion:**

Women’s empowerment has long been identified as being able to bring about progress in various areas, including health. It has been shown that men’s commitment to transforming gender norms is a sinequanone factor for greater equity and better health, especially in terms of reproductive health and child nutrition. This study is one of the first in this genre in DRC and results will serve as a guide for policies aimed at improving the involvement of men in changing attitudes towards gender norms for higher household productivity and better health.

## Background

Maternal and child health are among the most important indicators of a country’s development. Major causes of maternal mortality can be prevented and treated with simple, affordable health interventions such as antenatal care (ANC) or vaccination if used adequately and on time [1]. Children’s nutritional status is a sensitive indicator for household poverty, with childhood malnutrition having many long-term effects for health [2–4]. The Democratic Republic of the Congo (DRC) is among the poorest countries in the world and heavily indebted with high rates of maternal and child mortality [5]. The country was ranked as the 176th of 188 countries in terms of human development index [5]. In 2015 maternal mortality was estimated at 693 per 100,000 and the under-five mortality rate was around 308 per 1000 [6]. Malnutrition is rampant where 43% of children under five are malnourished and around 76% of women encounter barriers accessing maternal health care services [7]. The country has been involved in repetitive wars for over 20 years, and the East of the country is still facing rebellions. Insecurity prevails and unemployment is widespread. Like in many other countries, women ensure much of the economic activities of a household, and largely carry the responsibility for providing and caring for household members; men endorse the role of decision-making for all expenditures including health [8–10]. This is confirmed according to the Demographic and Health Survey 2014/15, where only 29% women decided themselves how to use the money they have earned. When it comes to decision making about their own health only 36% of the women decided themselves [7]. As women are usually the main caregivers of children, women’s limited decision-making negatively affects their children’s health and wellbeing [11–14]. Men’s involvement remains important though. A lack of participation of men in programs improving sexual and reproductive health has also been revealed as one of the reasons for poor progress observed in the domain of family planning and persisting disagreement between spouses regarding their choice for sexual and reproductive health service use [15–18]. Studies showed that male involvement led to better results regarding the use of contraceptives [18–20]. Barker and others have further demonstrated that the involvement of men in family health can be a mediator towards better health [21–24].

Women’s empowerment is a process of awareness and capacity building leading to greater participation, to greater decision-making power, and to transformative action in different domains such as rights, health, or economics [8, 25]. In the literature, economic empowerment was presented as a key factor for better health and to palliate all types of violence including intimate partner violence [26–28]. However, in a male-dominated society, women’s economic empowerment is not the sole factor to fight partner violence and can lead to resistance due to restrictive gender norms. Therefore, actions engaging men to support women’s empowerment must be part of interventions that aim at strengthening women’s economic participation [29–31] .

It has been shown that sufficient resources and an enabling environment allows women to make decisions about their own health, which leads to improved health outcomes specifically as regards sexual and reproductive health (SRH) [32–35]. Yet, in South Kivu and in other African contexts women often lack access to health services and also the financial resources to meet every day needs [9, 36]. As both are required, improvement of cash income, and a more equitable decision-making about the use of resources at the level of the household, complex interventions combining those two aspects are needed.

One of the most widely implemented approaches to improve population health is the introduction of microfinance including projects containing a social development component such as Village Savings and Loans Associations (VSLA) [37–40]. A VSLA is a platform for developing women’s capacities in organizational and financial matters, to improve self-efficacy and decision-making, and other skills. This approach was receiving increasing attention in the 1990s as a community-managed microfinance approach with a social mobilization component [41]. A VSLA is a self-selected group of 25 to 30 persons who agree to save a certain amount defined by all the members every week. Members self-organize in a committee with a president and meet every week. Loans can be taken up to 3 times the savings a member has contributed, and the loan has to be paid back with an interest rate of 5–10% according to what the group has defined. A normal cycle of a VSLA is nine to 12 months, after that a new VSLA has to be set up.

Several studies evaluated VSLA programs showing positive effects on general welfare and on child well-being [37, 39, 42]. Positive impacts on health were stronger if VSLA programs were in conjuncture with health education [21, 35, 43]. There is some evidence on the effectiveness of VSLAs in fragile contexts, which focused on specific subgroups. A study in post-conflict Ivory Coast showed positive effects of an intervention focusing on gender relations in combination with VSLAs on household economies and gender equity [29]. In Eastern DRC, a project evaluation among female sexual violence survivors showed a positive effect of a VSLA program on food consumption and stigma reduction [44]. A limitation of the approach is however the self-selection process when forming VSLA groups, which can lead to the exclusion of socially marginalized persons if they are not explicitly targeted [41, 45].

To improve gender equity within households, several approaches towards engaging men for gender equity have been developed, a prominent example being the Promundo approach, which is based on men-to-men sensitization developing ‘positive masculinity’. Men engage men towards more equitable gender norms to adopt attitudes and behaviour promoting women’s economic empowerment and helping to reduce gender based violence; “Positive masculinity” refers to positive changes in attitudes and behaviours transforming the socio-cultural norms associated with masculinity [46, 47]. Once men are sensitized to the benefits of women’s empowerment, they could become active advocates for women’s and children’s welfare in the household as well as in the community and change gender-based inequities in health [38].

Increasingly, projects target women and men jointly in order to transform gender-inequitable norms and behaviors, yielding encouraging results for example on reducing gender based violence [30, 48, 49]. Projects engaging men can be part of complex interventions, but only a few have combined these approaches with VSLAs [29, 31].

### Mawe tatu program

**Fig. 1:**
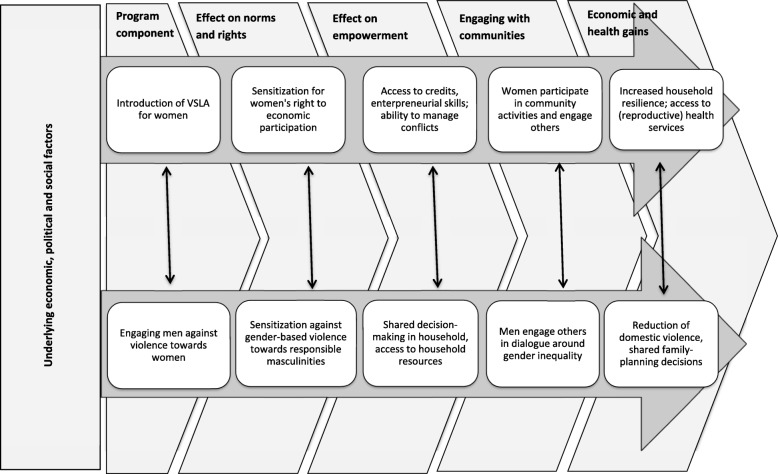
Mawe tatu’s Project framework

The “Mawe tatu” program, implemented in the Eastern part of the Democratic Republic of Congo, links VSLAs for women with men-to-men sensitization to transform gender-inequitable norms and behaviors, and additionally combines these two project arms with an educational component about family planning and sexual and reproductive health. The project is implemented by CARE international, with the three components linked to three main objectives:

1. empowerment of women by organizing them in Village Savings and Loans Associations (VSLA). An important component of a VSLA besides the financial aspect is the development of women’s capacities in organizational and financial matters, to improve self-efficacy and decision-making, and other skills. In addition, different discussion topics are developed during the cursus to further improve knowledge and skills: Human Right, Leadership, Governance, House Economics, Conflict Resolution, Family Planning, Gender Equity and Diversity.
2. Developing ‘positive masculinity’ by engaging men, if possible spouses of VSLA’s members, towards women’s rights using a peer-to-peer approach. Men should sensitize each other and organize themselves in reflection groups in order to adopt attitudes and behaviours promoting women’s economic empowerment and reducing gender based violence. Sensitisation includes the following themes: identity and the concepts of gender; masculinities; the cycle of male and female socialization; the socialization of gender: acting as a man, acting like a woman; men and interpersonal violence; gender-based violence (GBV); involvement of men in the prevention of GBV; the ten tips for a good fatherhood: sharing of work and child care within the household; fair negotiation, equity and equality; economic partnership; mutual respect and dialogue between spouses; family planning; dissemination methodology.
3. As a third component comprehensive sexuality education for young people, which includes gender and rights themes, is offered as well. The three approaches work in interaction sustaining women’s empowerment in order to increase gender equity, reduce poverty and improve health at the level of households and at the level of community (Fig. 1). The evaluation of the third component is not part of this protocol.

This research aims to clarify (a) the impact of a complex intervention associating VSLA program with men’s engage program on reproductive and child health indicators, and (b) to clarify the contribution of a peer-to-peer sensitization approach engaging men for more equitable gender norms as part of a complex intervention. Main outcomes include household economy, gender norms and gender-based violence, and women’s confidence to participate in household- and community-decision-making processes. Reproductive and child health outcomes include child nutritional status, utilization of antenatal care services, facility-based delivery, family-planning counselling, and women’s self-rated health (Fig. 1).

### Conceptual framework

**Fig. 2:**
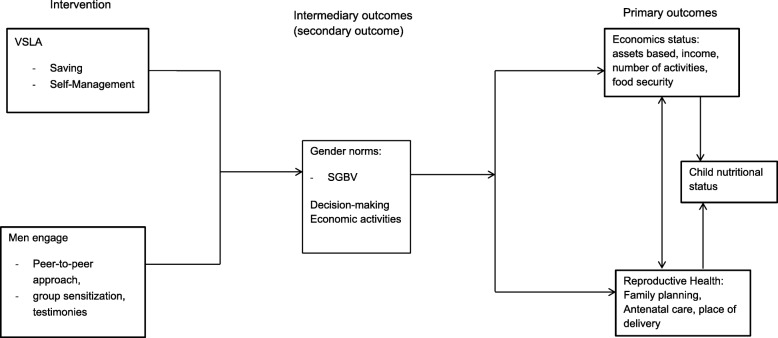
Research framework

A framework has been developed to operationalize how “positive masculinity” in combination with women’s economic empowerment impact on the economic status of the household and on health and wellbeing (Fig. 2).

Through the MaweTatu project, men are sensitized for women’s rights, including sexual and reproductive health rights. Together with women participating in VSLAs they are expected to become agents of change as regards the transformation of gender norms, which will lead to more gender-equitable attitudes, joint intra-household decision-making, and increased economic activities and autonomy among women. It is further expected that engaging men for gender equity enhances health and wellbeing of the family, including children’s nutritional status.

## Methods

This research will employ a parallel mixed-method design, combining a longitudinal cohort study (Study1) with a longitudinally designed qualitative study (Study 2). Quantitative findings will be triangulated with qualitative findings in order to deepen the understanding of the forces that trigger and sustain the expected change.

### Study component 1 - quantitative

#### Study design and study population

A cluster-randomized, longitudinal intervention study compares VSLA participants in an intervention area with controls over a period of 12 months. The intervention districts are identified a priori by the implementing organization, but the intervention and control sites (villages) are randomly selected within these districts. In the intervention sites, all persons participating in a newly created VSLA are eligible for inclusion in the intervention arm. While participation in a VSLA is based on self-selection, a random sample of VSLA participants is selected based on VSLA members’ lists available from the Mawe Tatu project.

A control group of participants is recruited in adjacent randomly selected villages where VSLAs are not offered. Participants self-select to take part in an information session on income-generation or a related theme in order to recruit participants with a similar socio-economic profile as the VSLA members. A random sample of participants of the information sessions is then included in the study as controls. In both the intervention and control areas, a community leader conveys the information about the upcoming activities in a similar way in order to attract a similar group of participants.

Additional inclusion criteria for the study included being long-term residents of the study site (living in the household for at least 6 months) and being at least 15 years old. For participants with children, all children aged 1–5 years currently living in the household of an adult study participant are recruited for inclusion in the anthropometric study module. Children included in the study must be under guardianship of the adult study participant (Table 1).

**Table 1:** Study population by intervention and control group, quantitative study component

Study participants	Intervention group	Control group
Women	VSLA member	Participated in information session, not member of a VSLA
Children (1–5 years)	Children who are in guardianship of an adult study participant member of VSLA	Children in guardianship of an adult participating in control group

#### Instruments

The survey questionnaire includes questions about primary outcomes: household economy (income-generation, income, assets, housing, relative household status, health insurance), child nutritional status; and unmet need of family planning. Secondary outcome variables encompass gender norms and rights (perception of women’s rights and gender equity, women’s participation in decision-making and income-generation, women’s utilization of reproductive health services, and women’s perceived self-efficacy to speak out in community meetings). Further questions include information about the household structure (household composition and headship),, and individual socio-demographic information (age, education, marital status, number of children), and program-related variables (participant of VSLA, time in VSLA, partner participation in men’s reflection groups) (Table 2). A locally adapted composite wealth score is calculated based on structure of the house, type of fuel for cooking, toilet facility, food security, and having a mobile phone or TV. Questions from previously validated instruments are used if available (Demographic and Health Survey [7], Food-insecurity experience scale [50], Gender-equitable Men scale [51]). Anthropometric data is collected from children under 5 years of age living in the household including weight, height and mid-upper arm circumference (MUAC). An overview over the indicators used is provided in Table 2.

**Table 2:** Study variables

Variables	Type of variable	Units
1. Primary outcomes		
Household economy		
Assets	Categorical	
Relative household economic status	Categorical	
Household food security	Likert scale, Qualitative data	1–3
Income-generating activities	Categorical	
Income	continuous	
Housing	categorical	
Composite wealth indicator, asset based	Categorical	
Child nutritional status		
Weight	Continuous	Kg
Height	Continuous	Cm
MUAC	Continuous	Mm
Weight for Height	categorical	
Weight for age	Categorical	
Height for age	categorical	
Unmet need of family planning		
Counselling and use of FP	Binary	Yes or no
2. Secondary outcomes		
perception of women’s rights and gender equity,, and	Likert scale, Qualitative data	
women’s participation in decision-making and income-generation,	Likert scale, Qualitative data	
women’s utilization of reproductive health services		
Attendance of ANC during last pregnancy	Binary Qualitative data	Yes or no
Place of delivery of the last child	Categorical	Hospital, health care facilities, home
women’s perceived self-efficacy to speak out in community meetings	Likert scale, Qualitative data	
3. General characteristics		
Sex	Binary	Male/female
Age	Continuous	Months
Marital status of parents/tutor	Categorical	Married, single, divorced,
Household size	Continuous	
Sex of main breadwinner	Binary	Male/female
4. Program-related variables		
Belonging to VSLA	Binary	Yes or no
Time in VSLA	Continuous	
Partner or husband engaged in peer-to-peer reflexion group	Binary	Yes or no

#### Sample size calculation/justification

The power calculation is based on the hypothesis that establishing savings and loan systems at the village level lowers the risk of stunting of children up to the follow-up. Improved child growth is a result of increased livelihood and food security that is sustainable over some time. The surveys are conducted in 80 villages with an average of 15 households per village (1200 households). Assuming an attrition rate of 30% during follow-up this results in a final analytic sample size of 800. The control group is planned to be smaller with 40 villages (600 households). Assuming the same attrition rate there must be 400 households recruited to participate in the final control arm.

As there are few data on the distribution of individual growth rates among children in the study area, we express the intervention effect in terms of a certain fraction of the standard deviation SD of individual growth rates. If the mean change in height during a given time period increases by z standard deviations as a consequence of the intervention, then this corresponds to a shift of the median of growth to the Φ (z)-quantile of the distribution in the control group (where Φ denotes the cumulative density function of the standard normal distribution. For instance, if z = 0.25, this corresponds to an intervention-related shift of the distribution whose new median is where the 60th percentile of the original distribution was.

#### Data collection

A team of local researchers fluent in the locally spoken languages is trained over a week in data collection methods, followed by a pilot study. Participation in the survey is voluntary and refusal to participate will have no repercussion whatsoever. The study information and consent forms are translated into local languages. Informed verbal and written consent is obtained from each individual prior to the beginning of data collection. Data will be collected strictly respecting confidentiality. No compensation will be offered in exchange for participation in the survey and no fees will be required from participants. The structured questionnaire is administrated using tablet technology and the Open Data Kit (ODK) software package. Data is stored on a secured server located at the Swiss Tropical and Public Health institute in Basel.

Anthropometric measurements of children are taken by trained surveyors using a weighing scale, a tape measure and a (Mid-upper arm circumference) MUAC measuring tape [52].

#### Statistical analysis plan

An intention-to-treat analysis comparing all persons who were initially participating in a VSLA with a control group will establish the effect of participating in the project on household economies (composite wealth score; number of income-generating activities), on child nutritional status (height for age z-score HAZ, weight for age z-score WAZ, and weight for height z-score WHZ, mid-upper-arm circumference), and on the use of family planning (current use of modern family planning method; unmet need for contraception) as the primary outcomes.

##### Primary outcomes

Household economic status will be assessed using an asset-based wealth score and number of income-generating activities. For the analysis and validation of scaling properties of the composite wealth score principal component analysis will be used.

To measure child nutritional status, measures of chronic and acute malnutrition will be used. Stunting (small-for-age) as measure of chronic malnutrition will be measured as height for age index z-scores (HAZ): a HAZ < − 2SD was defined as stunted, a HAZ between -2SD and − 3 SD was defined as moderate stunting and a HAZ < − 3 z-score was defined as severe stunting. Underweight will be measured as weight for age index z-scores (WAZ): a WAZ < − 2SD was defined as underweight, a WAZ between – 2SD and − 3 SD was defined as moderate underweight and a WAZ < − 3SD was defined as severe underweight. Wasting, measuring acute malnutrition as weight for height z-scores (WHZ) < − 2 SD, a WHZ between – 2SD and – 3SD as moderate wasting and a WHZ < −3SD as severe wasting. A MUAC < 115 mm will also define a severe malnutrition [52–54]. To measure food security, the FAO food insecurity experience scale is used [50]. Family planning use is measured as proportion of women currently using modern contraceptives, and as unmet need for family planning.

##### Secondary outcomes

In addition to the primary outcomes, secondary outcomes include changing gender norms (attitudes towards women’s rights, gender-based violence, women’s roles) and women’s empowerment (participation in the economy; self-efficacy to express their views; intra-household decision-making; use of health services; gender-based violence). (Table 3).

**Table 3:** Power calculations for child anthropometric data

Effect size	Shift of median to	ICC^a^	SD of random village effect	Child in every HH	Child in 50% HH
				Power	Power
0.27 SD	P61	0.01	0.1 SD	99%	86%
		0.02	0.14 SD	98%	84%
		0.03	0.18 SD	97%	83%
		0.04	0.21 SD	96%	81%
		0.05	0.23 SD	95%	79%
		0.10	0.33 SD	86%	–
0.25 SD	P60	0.01	0.1 SD	97%	80%
		0.02	0.14 SD	96%	78%
		0.03	0.18 SD	95%	77%
		0.04	0.21 SD	93%	75%
		0.05	0.23 SD	91%	73%
		0.10	0.33 SD	80%	–
0.2 SD	P58	0.01	0.1 SD	88%	–
		0.02	0.14 SD	84%	–
		0.03	0.18 SD	81%	–
		0.04	0.21 SD	78%	–
		0.05	0.23 SD	75%	–

Table 3 gives the achievable power for different effect sizes and intra class correlation coefficients. The expected power is given both under the assumption that a) a child under 5 years old will be found in each household, and b) that a child under 5 years old is found only in every other household. Intra-class coefficients reported in other African contexts range from 0.01 to 0.05

*SD* standard deviation of individual growth rates

^a^Proportion of variance explained by the factor village

First, descriptive statistics will be calculated for the primary and secondary outcome variables, and for socio-economic characteristics. Assets, number of income-generating strategies, food security and child nutritional status, use of family planning and other reproductive health services, and related knowledge, and prevalence of perceptions of gender relations (beliefs and attitudes), and prevalence of different levels of knowledge on sexual and reproductive health including use of family planning methods and existing services will be documented. For this purpose, percentages, means and standard deviations will be computed. To assess self-efficacy and decision-making power, indices will be built using Mokken analysis, which is a nonparametric procedure based on item-response theory that has been used to assess similar scales in previous studies [55, 56]. Differences between education level, rural and urban populations will be assessed statistically using Chi2 tests for different degrees of freedom for categorical variables, or using t-tests for continuous variables.

To assess the program effects, mixed-effect regression models will be run for each primary outcome variable to establish change in the outcome variables over time (baseline to endline) and by intervention and control group. The models will be adjusted for socio-economic confounders, and clustering will be considered at the level of villages. The same analysis as for primary outcomes will be conducted for secondary outcomes. In addition, the role of mediating factors will be investigated. It will be explored whether a change in the power of decision-making mediates changes in household economic status and use of family planning (FP). In addition, we will study whether children’s nutritional status improves if men are supportive of women’s economic activities. For this purpose structural equation modelling (SEM) with maximum likelihood estimation will be conducted to investigate the complex and multidimensional pathways by which the association of a positive masculinity and women’s empowerment directly or indirectly affect household economics status, children nutritional’s status and use of reproductive health services, and the potential role of mediating variables.

Results will be discussed under consideration of the fidelity of the intervention. Women who complete the full cycle of a VSLA and men who participate in the full cursus of the peer-to-peer sensitisation group will determine the level of fidelity.

Analysis will be conducted with STATA V.15.

### Study 2 - qualitative component

A qualitative study with households participating in a VSLA will be conducted, collecting data on gender relations, women’s economic participation and access to sexual and reproductive health services.

#### Study design

Qualitative studies are by nature smaller and capable of providing in-depth insights into processes within selected households and couples of a particular study site [57].

With the qualitative study, women and their partners are closely followed through multiple interviews, including collection of information related to their income and expenditure, as well as information on gender-based dynamics within families and communities. Health-related behaviour and perceptions of family planning will also be explored in the context of men engaged and women’s empowerment to assess a change in behaviours and perceptions after men’s sensitization as well motivations for the change.

#### Instruments

For the qualitative interviews a guide is developed focusing on the impact participation in the project has on women’s families in terms of gender-relations, household economy, and health. Over time, the instrument is adapted based on the results from previous interviews to capture emerging themes. Focus group discussions (FGD) with 6–8 participants, with women and men separately will be organized as well as individual in-depth interviews (IDIs). We will use purposive sampling to recruit 18–49 years old women participating in VSLA whose husbands are also participating in either VSLA or a reflection group. Two focus groups will be organized with the same participants after a one-year interval. We plan to do five to seven FGDs coupled with IDIs carried out with same participants as FGD.

#### Study population and sampling

Participants of the qualitative study are recruited from households where both partners are involved in the intervention: women participating in VSLA and their partner participating in a male reflection group. Women and men in qualitative are recruited in the same villages but are not part of the quantitative study. We plan to conduct 20–30 in depth interviews with women and men or until saturation is reached [58]. Different themes such as gender norms, roles and justice in society, communication between husband and wives, and gendered responsibility in health and in households’ economics will be explored. Observations of women in VSLAs and at their home during the visits will be done throughout the process. Attitudes, the ability to feel confident to speak about anything related to women’s health and the nutrition of children with special regards on the involvement of men will be collected.

#### Data collection

Interview guides will be developed to explore dynamics of participating in VSLA and men’s sensitization for traditional roles and decision making at the level of the household with a specific focus on gender, household economy, and maternal and children health. Interviews will be carried out by a researcher familiar with the local setting and language. Interviews will be conducted in Kiswahili after written informed consent is obtained. No compensation will be offered in exchange for participation in the study. Interviews will be conducted in a isolated place chosen by the participant either in their home or outside. The researcher will make sure that privacy and confidentiality is always granted. The narratives from qualitative data collection will be voice-recorded and transcribed in Swahili. Observations will also be done during VSLA discussion sessions; notes will be taken and transcribed in French language. VSLA discussions on health topics relevant to this study will eventuality be targeted and recorded for analysis. Observations of women in VSLAs and at their home during the visit will be conducted; notes will be taken and transcribed in French.

#### Data analysis plan

Coding will be done with the help of qualitative data analysis software (Atlas TI). Latent themes will be identified by inductive analysis, reading and re-reading transcripts as well as notes from observations. At every round of iterative analysis emergent codes will be compared, grouped and contextualized. Finally, using a hermeneutic approach, the emerging hypotheses will be integrated in a wider contextual analysis.

### Quantitative and qualitative: data triangulation

Quantitative data will provide associations between outcomes and different factors in the study. Throughout the analysis, qualitative and quantitative results will be discussed in the study team, and triangulation will be done between qualitative and quantitative results (convergent parallel design). The qualitative analysis will shed light on aspects that cannot easily be quantified. Qualitative research is needed in order to understand why people give a specific answer especially for sensitive topics like gender, power and decision-making as those are strongly linked to social norms and individuals perceptions. It will also help to generate hypotheses on how to construct the structural equation models during the quantitative analysis.

## Discussion

Women’s empowerment can bring about change in various areas, including health [8, 43, 59]. Nevertheless, it has been argued that this is not enough. It has been shown that men’s commitment to gender equity is an indispensable prerequisite for better results, especially in terms of reproductive health and child nutrition. This study of a complex intervention will attempt to verify the combination of the two: women’s empowerment on one side and peer-to peer sensitization among men on the other side. Following participants longitudinally will yield a comprehensive understanding of the effect of positive masculinity on the use of reproductive health services and the nutrition of children, and on gender-power dynamics at the household and community levels.

This study, the first of its kind in the region investigating the impact of men’s involvement on women’s health and child nutrition could serve as basis for further research, in particular to explore the impact of men’s involvement on maternal and infant morbidity and mortality. Results will also help to define how best to improve engaging men in the transformation of social and gender norms and hopefully to define new strategies for improving maternal and child health through community-based, participatory interventions. This study will serve as a guide for policies aimed at improving the involvement of men to improve women’s health by increasing the use of maternal health services (antenatal care, family planning, maternity) and to reduce children’s malnutrition.

## Data Availability

Dataset generated and/or analysed are not publicly available due to confidentiality and anonymity of study population. Datasets are stored on a secure Alfresco website and are available from the corresponding author on reasonable request.

## Abbreviations

ANC: AnteNatal Care
DRC: Democratic Republic of Congo
EDS: Enquête Démographique de Santé
EKNZ: Ethic Kommission Nord-und Zentralschweiz
FGD: Focus Group Discussion
HAZ: Height for Age (stunting)
IDIs: In-depth Interviews
MUAC: Mid up arm circumference
NGO: Non-governmental organization
SEM: Structural Equation Modeling
UNDP: United Nations Development and Population
VSLA: Villages save loans associations
WHO: World Health Organization
WHZ: Weight for Height (wasting)
